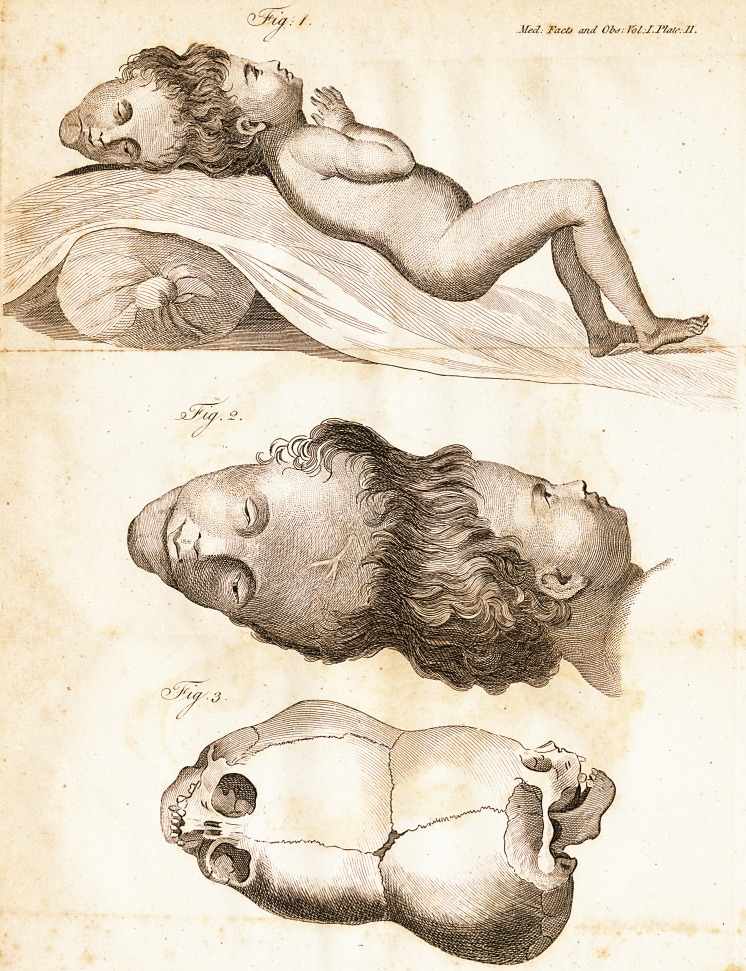# An Account of a Child with a Double Head

**Published:** 1791

**Authors:** Everard Home


XVII. *An Account of a Child with a double Head. In a Letter from* Everard Home, *Eſq. F. R. S. to* John Hunter, *Eſq. F. R. S.* — Vide *Philoſophical Tranſactions of the Royal Society of London,* Vol. LXXX. *for the Year* 1790, Part II.

THE ſpecies of *luſus naturæ*, which is the ſubject of the curious and intereſting paper before us, is ſo extraordinary and unaccountable, that, although the facts are ſufficiently eſtabliſhed by the teſtimonies of the moſt reſpectable witneſſes, the author would ſtill, as he very candidly aſſures us, have been diffident in bringing them before the Royal Society, had he not been enabled at the ſame time to produce the double ſkull itſelf, in which the appearances illuſtrate ſo clearly the different parts of the hiſtory, that it muſt be rendered perfectly ſatisfactory to the minds of the moſt incredulous.

The

Fig: 1.

Fig. 2.

Fig. 3.

The following account of the child, when ſix months old, he was favoured with by Sir Joſeph Banks, who, it ſeems, from the hand-writing and other circumſtances, believes that it was written by the late Colonel Pierce. Mr. Home has, however, he tells us, been leſs ſolicitous to aſcertain the author, as the obſervations contained in this account agree ſo entirely with the remarks that were afterwards made, and with the appearances of the ſkull, that they require no name being annexed to them in confirmation of their having been made with accuracy and fidelity.

"The child was born in May, 1783, of "poor parents; the mother was thirty years "old, and named Nooki; the father was called "Hannai, a farmer at Mandalgent, near Bar-"dawan, in Bengal, and aged thirty-five.

"At the time of the child's birth, the wo-"man who acted as midwife, terrified at the "ſtrange appearance of the double head, en-"deavoured to deſtroy the infant by throwing "it upon the fire, where it lay a ſufficient time, "before it was removed, to have one of the "eyes and ears conſiderably burnt.

"The body of the child was naturally form-"ed, but the head appeared double, there be-

" ing, befides the proper head of the child, " another of the fame fize, and to appearance " almoft equally perfect, attached to its upper " part. This upper head was inverted, fo that " they feemed to be two feparate heads united " together by a firm adhefion between their " crowns, but without any indentation at their " union, there being a fmooth continued fur- " face from the one to the other. The face of " the upper head was not over that of the " lower, but had an oblique pofition, the cen- " ter of it being immediately above the right " eye.

" When the child was fix months old, both " of the heads were covered with black hair " in nearly the fame quantity. At this period " the fkulls feemed to have been completely " offified, except a fmall fpace between the offa " frontis of the upper one, like a fontanelle.

" *Obfervations on the fuperior or inverted Head.*

" No pulfation could be felt in the fituation " of the temporal arteries; but the fuperficial " veins were very evident.

" The neck was about two inches long, and " the upper part of it terminated in a rounded " foft tumor, like a fmall peach.

" One

" One of the eyes had been confiderably " hurt by the fire, but the other appeared per- " fect, having its full quantity of motion; " but the eyelids were not thrown into action " by any thing fuddenly approaching the eye; " nor was the iris at thofe times in the leaft af- " fected, but, when fuddenly expofed to a " ftrong light, it contracted, although not fo " much as it ufually does. The eyes did not " correfpond in their motions with thofe of " the lower head; but appeared often to be " open when the child was afleep, and fhut " when it was awake.

" The external ears were very imperfect, " being only loofe folds of fkin, and one of " them mutilated by having been burnt. There " did not appear to be any paffage leading into " the bone which contains the organ of hear- " ing.

" The lower jaw was rather fmaller than it " naturally fhould be, but was capable of mo- " tion. The tongue was fmall, flat, and ad- " hered firmly to the lower jaw, except for " about half an inch at the tip, which was " loofe. The gums in both jaws had the na- " tural appearance; but no teeth were to be " feen either in this head or the other.

" The internal surfaces of the nose and

" mouth were lubricated by the natural secre-

" tions, a considerable quantity of mucus and

" saliva being occasionally discharged from

" them.

" The muscles of the face were evidently

" possessed of powers of action, and the whole

" head had a good deal of sensibility, since

" violence to the skin produced the distortion

" expressive of crying, and thrusting the fin-

" ger into the mouth made it shew strong

" marks of pain. When the mother's nipple

" was applied to the mouth, the lips attempted

" to suck.

" The natural head had nothing uncommon

" in its appearance; the eyes were attentive to

" objects, and its mouth sucked the breast vi-

" gorously. Its body was emaciated.

" The parents of the child were poor, and

" carried it about the streets of Calcutta as a

" curiosity to be seen for money; and to pre-

" vent its being exposed to the populace, they

" kept it constantly covered up, which was

" considered as the cause of its being emaciated

" and unhealthy."

The attention of the curious could not fail to be attracted by so uncommon a species of deformity; deformity ; and Mr. Stark, who was then reſident in Bengal, paid, we are told, particular attention to the appearances of the different parts of the double head, and endeavoured to aſcertain the mode in which the two ſkulls were united, as well as to diſcover the ſympathies which exiſted between the two brains. This gentleman, on his return to England, finding that Mr. Home was in poſſeſſion of the ſkull, and propoſed drawing up an account of the child, very obligingly favoured him with the reſult of his obſervations, and at the ſame time permitted him to have a ſketch taken from a very exact painting, made under his own inſpection, from the child while alive, by Mr. Smith, a portrait painter then in India. From this figure *, and two others †, which accompany

* See Plate II. Fig. 1.

† See Plate II. Fig. 2 and 3. In fig. 2 the double head is repreſented exactly half the natural ſize. One of the eyes of the upper face appears ſmaller or more contracted than the other; this is ſaid to be in conſequence of the injury it received when the child was thrown upon the fire. In this figure the ſuperficial veins upon the forehead of the upper head are very diſtinctly ſeen.—Fig. 3 is an exact repreſentation of the double ſkull, which is now in Mr. Hunter's collection, upon the ſame ſcale as fig. 2. Mr. Home obſerves of it, that it ſhows pany Mr. Home's account, and which we have taken the liberty to copy for the gratification of our readers, a very accurate idea is given of the child's appearance.

At the time Mr. Stark ſaw the child it muſt have been, our author thinks, nearly two years old *, as it was ſome months before its death, which he has every reaſon to believe happened in the year 1785. At this period the appearances, we are informed, differed in many reſpects from thoſe taken notice of when the child was only ſix months old.

The burnt ear had ſo much recovered itſelf as only to have loſt about one fourth part of the looſe pendulous flap. The openings leading from the external ear appeared as diſtinct as in thoſe of the other head. The ſkin ſurrounding the injured eye, which was on the ſame ſide

ſhows the curious manner in which the two ſkulls are united together, and the number of teeth formed before the child's death; which circumſtance, he adds, aſcertains with tolerable accuracy its age.

* Mr. Home remarks, in a note, that the dentes molares, which uſually appear at twenty months or two years of age, were through the gums; and there was no reaſon, he adds, to expect them very early in this child.

with with the mutilated ear, was in a flight degree affected, and the external canthus much contracted, but the eye itfelf was perfect.

The eyelids of the fuperior head were never completely fhut, remaining a little open, even when the child was afleep, and the eyeballs moved at random. When the child was roufed, the eyes of both heads moved at the fame time; but thofe of the fuperior head did not appear to be directed to the fame object, but wandered in different directions. The tears flowed from the eyes of the fuperior head almoft conftantly, but never from the eyes of the other, except when crying.

The termination of the upper neck was very irregular, a good deal refembling the cicatrix of an old fore.

The fuperior head feemed to fympathize with the child in its natural actions. When the child cried, the features of this head were affected in a fimilar manner, and the tears flowed plentifully. When it fucked the mother, fatisfaction was expreffed by the mouth of the fuperior head, and the faliva flowed more copioufly than at any other time; for it always flowed a little from it. When the child fmiled, the features of the fuperior perior head ſympathiſed in that action. When the ſkin of the ſuperior head was pinched, the child ſeemed to feel little or no pain, at leaſt not in the ſame proportion as was felt from a ſimilar violence being committed on its own head or body.

When the child was about two years old, and in perfect health, the mother, we are told, went out to fetch ſome water, and upon her return, found it dead, from the bite of a *Cobra de Capelo.* The body was buried near the banks of the Boopnorain river, but was afterwards dug up by Mr. Dent, the honourable Eaſt India Company's Agent for ſalt at Tumloch, on whoſe grounds the parents of the child then lived. By Mr. Dent it was given to Captain Buchanan, late Commander of the Ranger Packet, in the ſervice of the honourable the Eaſt India Company, who, being ſtruck with the uncommon appearance of the double ſcull, had expreſſed a wiſh that he might be permitted to bring it to Europe and preſent it to our author, to whom he well knew it would be highly acceptable. This requeſt, we are informed, was no ſooner communicated to Mr. Dent, than it was complied with; and Mr. Home obſerves that he ſhould do both theſe gentlemen gentlemen injuftice, were he not to attribute their readinefs upon the prefent occafion to oblige him, in a great meafure to their knowing that the double fkull would be depofited in Mr. Hunter's Collection, which muft now be confidered more as a national and public repofitory than a private cabinet.

Mr. Home remarks that the two fkulls which compofe this monftrous head appear to be nearly of the fame fize, and equally complete in their offification, except a fmall fpace at the upper edge of the offa frontis of the fuperior fkull; fimilar to a fontanelle. The mode, he tells us, in which the two were united is curious, as no portion of bone is either added or diminifhed for that purpofe; but the frontal and parietal bones of each fkull, inftead of being bent inwards, fo as to form the top of the head, are continued on; and, from the oblique pofition of the two heads, the bones of the one pafs a little way into the natural futures of the other, forming a zig-zag line, or circular future, uniting them together.

The two fkulls are faid to be almoft equally perfect at their union; but the fuperior fkull, as it recedes from the other, is defcribed as becoming more imperfect and deficient in many of its parts. Mr. Home obſerves, for inſtance, that the meatus auditorius in the temporal bone is altogether wanting; and that the baſis of the ſkull is imperfect in ſeveral reſpects, particularly in ſuch parts as are to connect the ſkull with a body, the foramen magnum occipitale being only a ſmall irregular hole, very inſufficient to give paſſage to a medulla ſpinalis, and there being no condyles with articulating ſurfaces round its margin, as there were no vertebræ of the neck to be attached to it. He farther remarks that the foramen lacerum in the baſis of the cranium is only to be ſeen on one ſide, and even there is too ſmall for the jugular vein to have paſſed through; that the oſſa palati are deficient at their poſterior part; that the lower jaw is too ſmall for the upper; and that the condyle and coronoid proceſs of one ſide are wholly wanting.

In moſt other reſpects, the two ſkulls, we are told, are alike; the number of teeth in both being the ſame, viz. ſixteen.

From an examination of the internal ſtructure of the double ſkull, the two brains, our author obſerves, have certainly been incloſed in one bony caſe, there being no ſeptum of bone between them. How far they were intirely diſtinct, and ſurrounded by their proper membranes cannot now be aſcertained; but from the ſympathies which were taken notice of by Mr. Stark, between the two heads, more particularly thoſe of the ſuperior with the lower, or more perfect, Mr. Home is inclined to believe, that there was a more intimate connexion between them than ſimply by means of nerves, and therefore that the ſubſtance of the brains was continued into one another.

Had the child, he obſerves, lived to a more advanced age, and given men of obſervation opportunities of attending to the effects of this double brain, its influence upon the intellectual principle muſt have afforded a curious and uſeful ſource of inquiry; but unfortunately, he adds, the child only lived long enough to complete the oſſification of the ſkull ſo as to retain its ſhape, by which means he has been enabled to aſcertain and regiſter the fact, without having enjoyed the ſatisfaction that would have reſulted from an examination of the brain itſelf, and a more mature inveſtigation of the effects it would have produced.

## Figures and Tables

**Fig: 1. Fig. 2. Fig. 3. f1:**